# Antimicrobial resistance in commensal *Escherichia coli* from humans and chickens in the Mekong Delta of Vietnam is driven by antimicrobial usage and potential cross-species transmission

**DOI:** 10.1093/jacamr/dlac054

**Published:** 2022-05-27

**Authors:** Nguyen Thi Nhung, Nguyen Thi Phuong Yen, Nguyen Thi Thuy Dung, Nguyen Thi Minh Nhan, Doan Hoang Phu, Bach Tuan Kiet, Guy Thwaites, Ronald B. Geskus, Stephen Baker, Juan Carrique-Mas, Marc Choisy

**Affiliations:** Oxford University Clinical Research Unit, Ho Chi Minh City, Vietnam; Oxford University Clinical Research Unit, Ho Chi Minh City, Vietnam; Oxford University Clinical Research Unit, Ho Chi Minh City, Vietnam; Oxford University Clinical Research Unit, Ho Chi Minh City, Vietnam; Oxford University Clinical Research Unit, Ho Chi Minh City, Vietnam; Faculty of Animal Science and Veterinary Medicine, Nong Lam University, Ho Chi Minh City, Vietnam; Sub-Department of Animal Health and Production, Dong Thap Province, Vietnam; Oxford University Clinical Research Unit, Ho Chi Minh City, Vietnam; Centre for Tropical Medicine and Global Health, University of Oxford, Oxford, UK; Oxford University Clinical Research Unit, Ho Chi Minh City, Vietnam; Centre for Tropical Medicine and Global Health, University of Oxford, Oxford, UK; Cambridge Institute of Therapeutic Immunology & Infectious Disease, University of Cambridge, Cambridge, UK; Oxford University Clinical Research Unit, Ho Chi Minh City, Vietnam; Centre for Tropical Medicine and Global Health, University of Oxford, Oxford, UK; Oxford University Clinical Research Unit, Ho Chi Minh City, Vietnam; Centre for Tropical Medicine and Global Health, University of Oxford, Oxford, UK

## Abstract

**Objectives:**

To investigate phenotypic antimicrobial resistance (AMR) in relation to antimicrobial use (AMU) and potential inter-species transmission among *Escherichia coli* from humans and chickens located in the same households in the Mekong Delta of Vietnam.

**Methods:**

We collected data on AMU and faecal swabs from humans (*N *= 426) and chickens (*N *= 237) from 237 small-scale farms. From each sample, one *E. coli* strain was isolated and tested for its susceptibility against 11 antimicrobials by Sensititre AST. The association between AMR and AMU was investigated by logistic regression modelling. Using randomization, we compared the degree of similarity in AMR patterns between human and chicken *E. coli* from the same farms compared with isolates from different farms.

**Results:**

The AMU rate was ∼19 times higher in chickens (291.1 per 1000 chicken-days) than in humans (15.1 per 1000 person-days). Isolates from chickens also displayed a higher prevalence of multidrug resistance (63.3%) than those of human origin (55.1%). AMU increased the probability of resistance in isolates from human (ORs between 2.1 and 5.3) and chicken (ORs between 1.9 and 4.8). *E. coli* from humans and chickens living on same farms had a higher degree of similarity in their AMR patterns than isolates from humans and chicken living on different farms.

**Conclusions:**

We demonstrated the co-influence of AMU and potential transmission on observed phenotypic AMR patterns among *E. coli* isolates from food-producing animals and in-contact humans. Restricting unnecessary AMU alongside limiting interspecies contact (i.e. increasing hygiene and biocontainment) are essential for reducing the burden of AMR.

## Introduction

A recently published systematic analysis estimated there were 4.95 million deaths worldwide in 2019 that were associated with antimicrobial resistance (AMR).^1^ Resistant bacteria arising either in humans, animals or the environment may spread between these three compartments.^2,3^ There is a growing consensus that adopting a One Health approach is imperative to improve our understanding of the epidemiology of AMR.^4,5^  *Escherichia coli*, often used as an indicator organism in AMR surveillance studies worldwide,^6–8^ is a major reservoir of AMR determinants that can be transferred horizontally between organisms of the same or closely related species.^9^

Demonstrating transmission of AMR between livestock and humans is challenging given the almost infinite combinations of antimicrobials, bacterial species, mobile genetic elements and host species, each having their own dynamics.^10^ Studies of humans and their livestock have demonstrated that bacteria from humans and animals are genetically linked^11^ and that exposure to animals harbouring MDR bacteria increased the probability of carrying MDR organisms in humans.^12^ A study in the Mekong Delta (Vietnam) showed an increased risk of colonization with *mcr*-1-carrying bacteria among farmers exposed to *mcr*-1-positive chickens.^13^ Conversely, there is limited or no evidence on the contribution of chicken farming to colonization with ESBL-producing bacteria in humans.^14,15^

Globally, antimicrobial consumption in humans has increased from 9.8 to 14.1 DDDs per 1000 inhabitants per day between 2000 and 2018, primarily driven by changes in low- and middle-income countries (LMICs).^16^ In some countries, antimicrobials are widely used in animal farming to treat infections, prevent diseases, and promote growth.^17^ Recent studies have shown that chicken flocks in the Mekong Delta were dosed with antimicrobials at a rate three times higher than the global average.^18^ The types of antimicrobials used are also a concern given that >50% of antimicrobial products intended for poultry contained one highest-priority critically important antimicrobial active ingredient.^19,20^

A meta-analysis has estimated that the overall prevalence of MDR in *E. coli* from healthy individuals in LMICs was ∼27%.^21^ A study in Ho Chi Minh City (southern Vietnam) reported that 71.8% of *E. coli* strains from healthy adults were MDR, and 9.7% were ESBL producers.^22^ Studies in the Mekong Delta have shown an overall high prevalence of resistance to critically important antimicrobials (CIAs) among chicken *E. coli* isolates, most notably to gentamicin (10.8%–42.2%), ciprofloxacin (21.0%–73.3%)^23–25^ and colistin (19.4%–22.2%).^23,26^ In contrast, a low prevalence of ESBLs (0.4%) was observed among *E. coli* isolates.^25^

There is now undisputed evidence that antimicrobial use (AMU) is a key driver for AMR both in community and hospital settings^27,28^ as well as in animal populations.^29^ Field trials on chicken flocks have also unequivocally demonstrated increases in diversity of AMR patterns among *E. coli* shortly after treatment.^30–32^ In Vietnam, studies on small-scale chicken flocks have demonstrated associations between usage of and resistance to ciprofloxacin and colistin.^13,23,25^ Establishing a clear relationship between the use of specific antimicrobials and resistance is challenging because of cross-resistance, a phenomenon by which certain antimicrobials can induce resistance to other, unrelated antimicrobials. Mechanisms such as multidrug efflux pumps or the co-location of resistance genes on a plasmid are partly responsible for the decreased susceptibility to multiple antimicrobials.^33,34^

Using a One Health approach, we sampled humans and chickens from smallholder farms in the Mekong Delta. The aims were (1) to describe phenotypic AMR of chicken and human *E. coli*; (2) to investigate the contribution of AMU on the observed AMR; and (3) to investigate the degree of similarity in the AMR profiles between *E. coli* from human and chickens on the same farms.

## Materials and methods

### Ethics

Informed consent was obtained from all participants. This study was granted ethics approval by the Oxford University Ethics Committee (OxTREC, Ref. No. 503-20).

### Study population

The study was conducted in Dong Thap Province, Mekong Delta of Vietnam (human population 1.6 million in 2019;^35^ chicken population 1.81 million^36^). We aimed to recruit 400 representative household farms raising chickens and a total of 800 human residents of these farms. Farm recruitment was conducted using a cluster sampling technique: 20 communes (across eight districts) were first selected, then, from each commune, a total of 20 chicken farms were recruited. All the farms raising chickens in flocks of >20 birds were eligible to join the study. All farm visits were carried out during June–July 2020.

### Data and sample collection

Staff affiliated to the Dong Thap Sub-Department of Animal Health and Production (SDAH-DT) collected chicken samples and farming-related data. Collection of human data and samples was conducted by the Dong Thap Center for Disease Control (CDC-DT) staff. Data on AMU and other variables were collected using structured questionnaires aimed at the person with primary responsibility for animal husbandry (Tables S1 and S2, available as Supplementary data at *JAC* Online). Interviewees were also asked to provide evidence (packages, bottles, sachets, etc.) of antimicrobial products used in their families and chicken flocks over the previous 90 and 7 days, respectively.

In each household, individual rectal swabs were collected from 1–3 people. A pooled faecal sample was collected by using a cotton swab and swabbing several points in the chicken pens where fresh droppings had been deposited. The swabs were stored at 4°C in brain–heart infusion broth (Oxoid, UK) plus 20% glycerol (Sigma, USA), then transferred to the laboratory and cultured within 24 h. All data were entered into a database using a web-based application.

### Laboratory processing of samples

From each sample solution, a volume of 10 μL was cultured on ECC agar (CHROMagar, France), a chromogenic medium allowing the detection of *E. coli*. One colony per sample showing typical *E. coli* morphology was selected. *E. coli* isolates were tested for their susceptibility to 11 antimicrobials belonging to eight classes by Sensititre AST (Thermo Fisher Scientific, UK) comprising colistin (polymyxins), cefpodoxime and ceftiofur (third-generation cephalosporins), azithromycin (macrolides), neomycin and streptomycin (aminoglycosides), amoxicillin (penicillins), enrofloxacin (quinolones), florfenicol (phenicols), doxycycline and oxytetracycline (tetracyclines). Potential production of ESBLs, as indicated by resistance to ceftiofur and/or cefpodoxime, was confirmed by double disc diffusion test according to the CLSI guidelines.

### Data analyses

Interpretation of the susceptibility status of tested strains was based on MIC breakpoint guidelines provided by CLSI, and National Antimicrobial Resistance Monitoring System for Enteric bacteria (NARMS) (Table S3). Intermediate-resistant isolates were regarded as resistant for analyses. MDR was defined as resistant to at least three different antimicrobial classes. The prevalences of resistance in human and chicken isolates were compared using Chi-square (*χ*^2^) tests for independence.

The usage rate per 1000 person-days or chicken-days was estimated using a Poisson regression model with the number of days when antimicrobials were consumed as the outcome, and the log-transformed observed duration as an offset. Quasi-likelihood was used to account for potential over-dispersion. When estimating the proportion of individuals using antimicrobials, we assumed that, among those declaring having used drugs, the proportions using antimicrobials were the same for those remembering and not remembering whether these drugs contained antimicrobials.^37^ In cases where individuals reporting using antimicrobials did not remember the duration of use, we assigned the average of the declared durations for the reported antimicrobials.

The association between AMU and AMR was investigated by logistic regression. For each antimicrobial tested, we compared the odds of resistance for individuals/birds exposed to antimicrobial versus those not having consumed any antimicrobial. Here, individuals/flocks that did not remember having used antimicrobial or not were excluded from the analyses. The exposures considered were: (1) use of target antimicrobial (i.e. same as the antimicrobial tested); (2) use of antimicrobials of the same class as the antimicrobial tested; (3) use any antimicrobial belonging to classes other than the antimicrobial tested; and (4) use of any antimicrobial. The presence of animals (other than chickens) on farms as well as recent AMU in these species were included in each model as covariates.

In order to investigate overall similarity of AMR profiles between *E. coli* isolates from humans and chickens we conducted discriminant analysis of principal components (DAPC) using the adegenet R package.^38^ In addition, we identified the most common pairs, triads, tetrads, pentads and hexads of AMR among all tested isolates by performing an agglomerative hierarchical clustering of the presence of resistance using the Jaccard distance metric (for binary data) with the average agglomeration method.^39^

We also investigated whether strains from humans and chickens on the same farm were more likely to be similar than those from different farms, as well as whether isolates from humans were more likely to be similar if collected from the same household than if collected from different households. The comparisons were performed for: (1) the presence of the resistance to individual antimicrobials; (2) the presence of the most common AMR clusters (pairs, triads, tetrads, pentads and hexads) identified by hierarchical clustering; (3) the whole pattern of resistance (both susceptible and resistant were considered). Comparisons of within versus between farms were performed by randomization of farm identity.^40^ For (1) and (2), the statistic used was the proportion of isolates from humans and chickens (or humans and humans) harbouring the same resistant cluster. For (3), the statistic used was the average number of matching antimicrobials with regards to the resistant/susceptible status of the isolates (11 antimicrobials). For instance, the number of matching antimicrobials (underlined) between the RSSRRSRRSSR and RSSSRSRRSRS resistance patterns is 8.

## Results

### Description of study farms

A total of 237 small-scale chicken farms were recruited; 426 residents from 233 of these farms consented to provide demographic and AMU information. Each farm had a median of 2 (first–third quartile 1–2) residents with a median age of 49 (first–third quartile 38–60) years-old. Collected data of human participants are presented in Tables S4 and S5 and their demographic characteristics are displayed in Table S6.

The median number of chickens per farm was 60 (first–third quartile 40–100) with the median age was 27.7 (first–third quartile 12–53) weeks. At the visit time, 86.9% (*n *= 206) of farms raised only one chicken flock, 11.8% (*n *= 28) raised two flocks and 0.5% (*n *= 3) raised three flocks. Of the study farms, 57.0% raised only chickens, and the remainder (43.0%) also raised other livestock (pigs or ducks or both). The majority of farms (81.0%) raised chickens for meat (with or without other purposes), and the remaining (19.0%) raised chickens for breeding or fighting or both. Data collected for chickens from farms are presented in Table S7 and S8.

### Antimicrobial use in humans and chickens

Of the 272 (63.8%) participants reporting using medicines over the last 90 days, 44 (16.2%) used antimicrobials. For the 95 participants reporting using medicines but being unsure whether medicines contained antimicrobials or not, we assumed that 16.2% of them (15 individuals) used antimicrobials. Therefore, antimicrobial use over the last 90 days were assumed for 59 (of 426) individuals (13.8%). A total of 34 different antimicrobials products (containing 19 antimicrobials belonging to seven classes) were identified.

Of the 127 (53.6%) farms reporting having administered medicines to chicken flocks, 104 (81.9%) used antimicrobials; among the owners of 12 flocks who were unsure whether medicines contained antimicrobials or not, we assumed that 10 of them (81.9%) used antimicrobials. Therefore, antimicrobials were administered to 114 (48.1% of 237) flocks. A total of 112 different antimicrobial products were identified, of which 52 (46.4%) contained only one antimicrobial and 60 (53.6%) contained two antimicrobials. A total of 30 antimicrobials belonging to 12 classes were identified.

Among 59 individuals who used antimicrobials, we assigned a duration of 9 days to 15 persons whose number of days of use was missing. Similarly, among 114 farms having administered antimicrobials to their chickens, the duration of 4 days was assigned to 13 flocks where owners did not remember the number of days of use. The rates of AMU per 1000 person- or chicken-days among human participants and chicken flocks are shown in Table 1. Overall, the AMU rate in chickens [291.1 (95% CI 248.9–340.8) per 1000 chicken-days], was about 19 times higher than that for humans [15.1 (95% CI, 10.2–22.4) per 1000 person-days, *P *< 0.001].

**Table 1.** Rate of antimicrobial use among humans and chickens^a^

Antimicrobial	Humans (*n *= 426)		Chickens (*n *= 237)		*P* value	*r*_c_/*r*_h_
	*N*	Rate per 1000 person-days (95% CI)	*N*	Rate per 1000 chicken-days (95% CI)		
**Cephalosporins**	**21**	**4.1 (2.4–7.3)**	**3**	**4.8 (1.4–16.7)**	**0.893**	**1.2**
***First- and second-gen. cephalosporins***	***16***	***2.6 (1.5–4.3)***	***2***	***4.2 (1.0–17.1)***	***0.602***	***1.6***
Cefadroxil	7	1.0 (0.4–2.1)	1	1.8 (0.2–12.8)	0.631	1.9
Cefalexin	5	0.7 (0.3–1.9)	1	2.4 (0.3–17.1)	0.348	3.2
Cefaclor	1	0.1 (0–0.9)				
Cefuroxime	5	0.7 (0.3–1.6)				
***Third-gen. cephalosporin***s**	***6***	***1.6 (0.5–4.4)***	***1***	***0.6 (0.1–4.3)***	***0.779***	***0.4***
Cefixime	4	1.5 (0.5–4.6)				
Cefdinir	1	0.2 (0–1.3)				
Cefpodoxime	1	0.03 (0–0.2)				
Ceftiofur			1	0.6 (0.1–4.3)		
**Aminocyclitols**			**20**	**31.9 (19.1–53.5)**	**NC**	
Spectinomycin			20	31.9 (19.1–53.5)		
Aminoglycosides*			**19**	**30.7 (17.1–55.3)**	**NC**	
Gentamicin			12	29.5 (15.9–54.7)		
Streptomycin			4	7.2 (2.1–25.0)		
Neomycin			3	10.2 (3.3–32.1)		
Tobramycin			1	1.2 (0.2–8.6)		
**Ansamycins**	**1**	**2.3 (0.3–16.7)**			**NC**	
Rifampicin	1	2.3 (0.3–16.7)				
Quinolones**	**8**	**1.4 (0.7–2.8)**	**14**	**30.7 (17.1–55.3)**	**<0.001**	**21.9**
Ciprofloxacin	3	0.5 (0.1–1.5)				
Levofloxacin	3	0.5 (0.2–1.7)				
Ofloxacin	2	0.4 (0.1–1.5)				
Enrofloxacin			12	29.5 (16.1–54.3)		
Norfloxacin			2	1.8 (0.4–7.8)		
Marbofloxacin			1	0.6 (0.1–4.3)		
**Folate pathway inhibitors**			**3**	**6.6 (1.7–26.0)**	**NC**	
Trimethoprim			3	6.6 (1.7–26.0)		
**Lincosamides**			**26**	**48.2 (31.1–74.8)**	**NC**	
Lincomycin			26	48.2 (31.1–74.8)		
Macrolides**	**4**	**0.8 (0.3–2.2)**	**29**	**76.5 (52.6–111.3)**	**<0.001**	**9.2**
Azithromycin	2	0.5 (0.1–2.1)				
Clarithromycin	1	0.2 (0–1.3)				
Erythromycin	1	0.2 (0–1.3)	6	12.7 (5.1–31.2)	<0.001	69.3
Tylosin			16	38.0 (22.4–64.3)		
Tilmicosin			7	18.7 (8.4–41.3)		
Spiramycin			2	8.4 (2.1–33.6)		
Penicillins*	**23**	**4.3 (2.6–7.2)**	**17**	**48.8 (29.3–81.2)**	**<0.001**	**11.3**
Amoxicillin	17	3.3 (2.0–5.3)	8	18.7 (8.3–42.2)	<0.001	5.7
Ampicillin	5	0.1 (0–0.3)	10	29.5 (15.3–57.0)	<0.001	226.5
Penicillin	1	0.8 (0.1–5.5)	1	0.6 (0.1–4.3)	0.953	0.8
**Phenicols**			**35**	**77.7 (54.2–111.5)**	**NC**	
Florfenicol			29	68.1 (45.6–101.7)		
Thiamphenicol			7	9.6 (4.3–21.5)		
Polymyxins**			**13**	**44.0 (25.1–77.0)**	**NC**	
Colistin			13	44.0 (25.1–77.0)		
**Sulphonamides**			**11**	**22.3 (11.2–44.3)**	**NC**	
Sulfadimidine			5	13.3 (4.9–35.5)		
Sulfamethoxazole			4	7.8 (2.8–21.9)		
Sulfamonomethoxine			2	2.4 (0.5–11.3)		
Sulfamethoxypyridazine			1	0.6 (0.1–4.3)		
**Tetracyclines**	**1**	**0.5 (0.1–3.9)**	**62**	**156.1 (121.3–201.0)**	**<0.001**	**285.0**
Doxycycline	1	0.5 (0.1–3.9)	28	67.5 (44.9–101.5)	<0.001	123.2
Oxytetracycline			33	86.8 (60.7–124.1)		
Tetracycline			8	22.3 (10.2–48.8)		
**Nitroimidazoles**	**2**	**0.4 (0.1–1.5)**			**NC**	
Metronidazole	2	0.4 (0.1–1.5)				
**Any antimicrobial**	**59**	**15.1 (10.2–22.4)**	**114**	**291.1 (248.9–340.8)**	**<0.001**	**19.2**

*N*, number using antimicrobials; NC, not calculated; CI, confidence interval; *r*_c_, AMU rate in chickens; *r*_h_, AMU rate in humans.

a Critically important antimicrobials according to WHO (2018) are marked with ** (highest priority) and * (high priority).

Among human participants, penicillins were used most [5.6% individuals, rate 4.3 (95% CI, 2.6–7.2)] followed by first-, second- and third-generation cephalosporins [4.9%, rate 4.1 (95% CI, 2.4–7.3)]. In contrast, the most frequently used antimicrobials among chicken flocks were tetracyclines [26.2% farms, rate 156.1 (95% CI, 121.3–201.0)] and phenicols [14.8% farms, rate 77.7 (95% CI, 54.2–111.5)]. Macrolides, quinolones, colistin, and ceftiofur (highest priority CIAs) had been given to chickens on 12.2%, 5.9%, 5.5% and 0.4% of farms, respectively.

### Phenotypic AMR in E. coli strains

**Figure 1.**
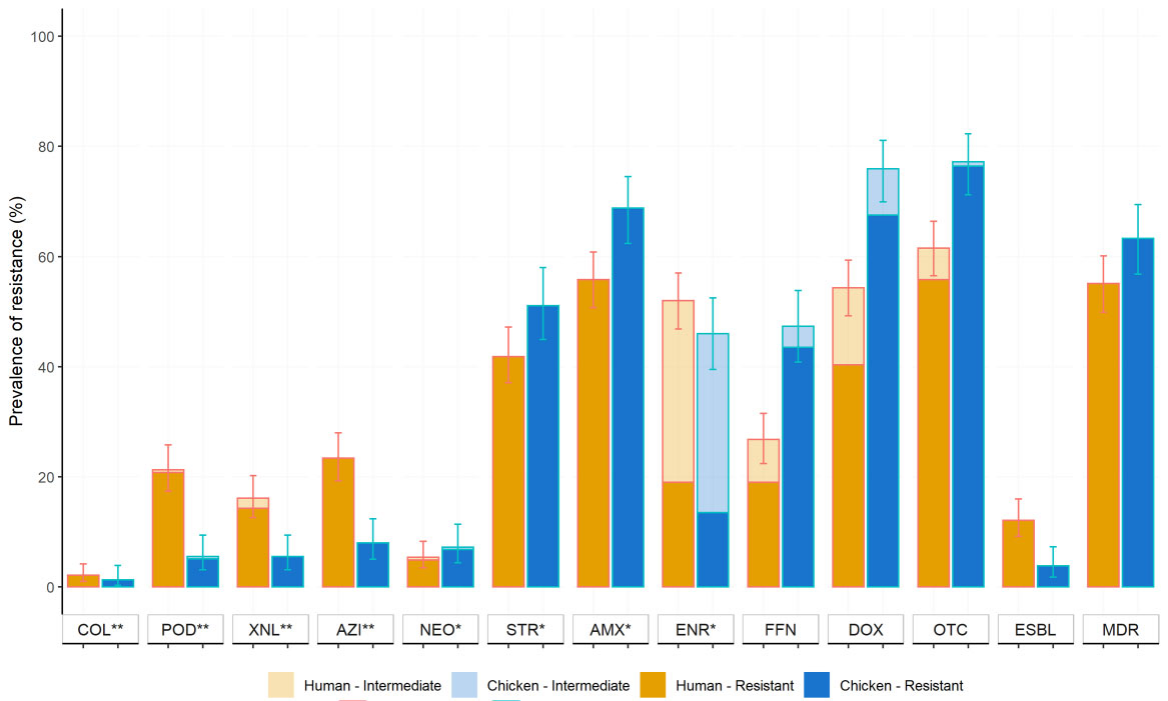
Prevalence of antimicrobial resistance among *E. coli* from humans (*n* = 385) and chickens (*n* = 237). COL, colistin; POD, cefpodoxime; XNL, ceftiofur; AZI, azithromycin; NEO, neomycin; STR, streptomycin; AMX, amoxicillin; ENR, enrofloxacin; FFN, florfenicol; DOX, doxycycline; OTC, oxytetracycline; ESBL, extended spectrum β-lactamase; MDR, multidrug resistance. Critically important antimicrobials according to WHO (2018) are marked with ** (highest priority) and * (high priority).

Of 426 human participants, 397 (from 229 households) consented to provide a faecal swab. Chicken faecal samples were collected from all study farms. A total of 385 and 237 *E. coli* strains were isolated from humans and chickens. Phenotypic resistance of *E. coli* strains is presented in Figure 1. Among the tested isolates, 311 (80.8%) from humans and 195 (82.3%) from chickens were resistant to at least one antimicrobial (*χ*^2^ test, *P *= 0.719). Chicken isolates displayed a higher prevalence of MDR (63.3%) compared with human isolates (55.1%, *χ*^2^ test, *P *= 0.053).

Compared with chicken *E. coli*, human isolates had higher levels of resistance to azithromycin (23.4% versus 8.0%), cefpodoxime (21.6% versus 5.5%), and ceftiofur (16.1% versus 5.5%) (χ^2^ test, all *P < *0.001). An ESBL phenotype was observed in 12.2% and 3.8% of human and chicken isolates, respectively (χ^2^ test, *P *< 0.001). In contrast, chicken isolates displayed a higher prevalence of resistance to oxytetracycline (77.2% versus 61.6%, *P *< 0.001), doxycycline (75.9% versus 54.3%, *P *< 0.001), amoxicillin (68.8% versus 55.8%, *P *= 0.002), and florfenicol (47.3% versus 26.6%, *P < *0.001). Colistin resistance among human and chicken *E. coli* was 2.1% and 1.3%, respectively (*χ*^2^ test, *P *= 0.665).

### Diversity of phenotypic AMR patterns

**Figure 2.**
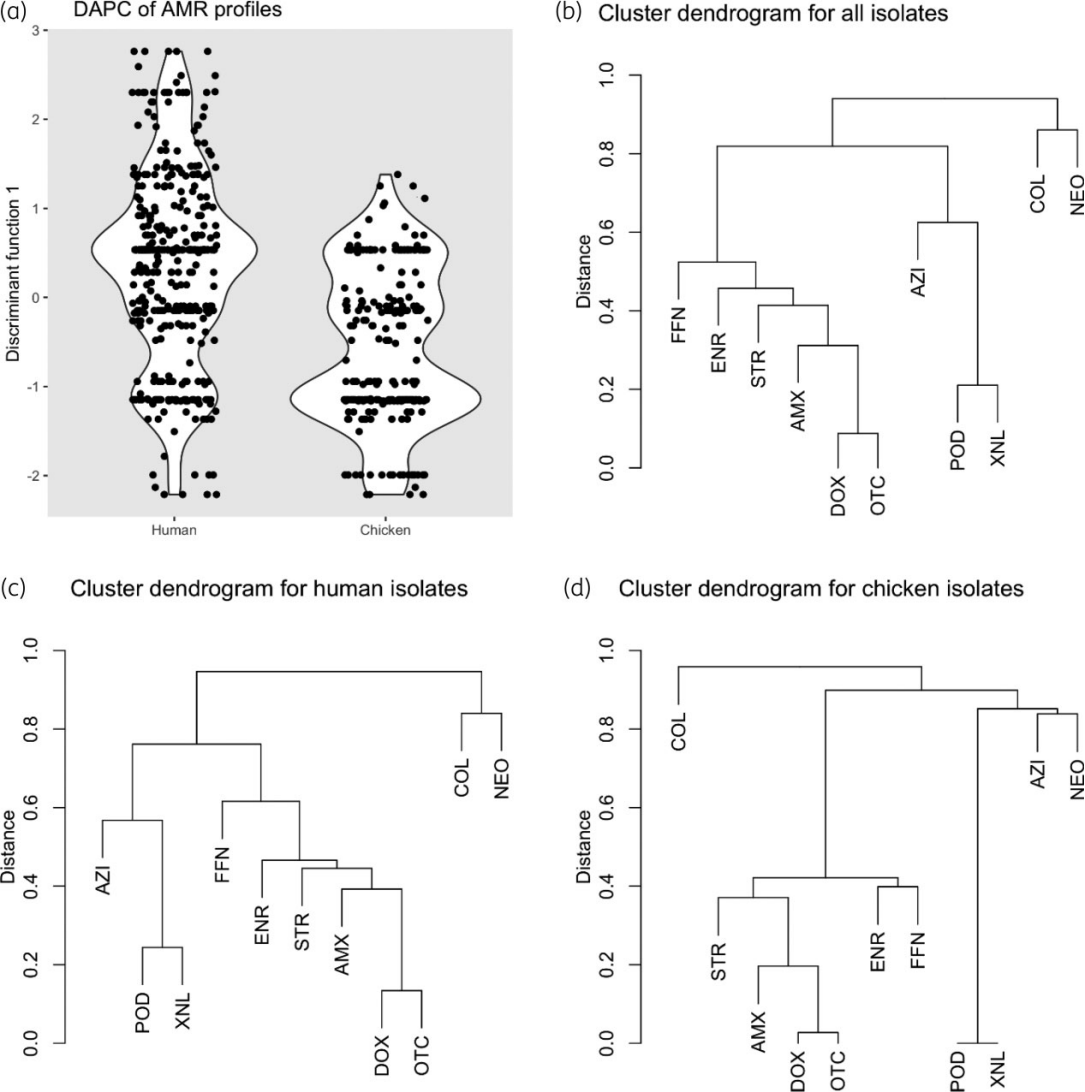
Representation of discriminant analysis of principal components (DAPC) of AMR profiles (a) of human (*n *= 385) and chicken (*n *= 237) isolates (each dot represents an isolate) and AMR cluster dendrograms of all (b), human (c) and chicken (d) isolates. COL, colistin; POD, cefpodoxime; XNL, ceftiofur; AZI, azithromycin; NEO, neomycin; STR, streptomycin; AMX, amoxicillin; ENR, enrofloxacin; FFN, florfenicol; DOX, doxycycline; OTC, oxytetracycline; MDR, multidrug resistance.

Overall, 110 different AMR patterns (to 11 antimicrobials) were identified, 103 in human and 44 in chicken isolates. DAPC analysis showed overall similarity in AMR pattern between isolates from humans and chickens (Figure 2a). Thirty-seven patterns were shared by isolates from two hosts (Table S9). Combined resistance to amoxicillin, doxycycline, enrofloxacin, florfenicol, oxytetracycline and streptomycin was the most common AMR pattern among both human and chicken isolates (8.1% and 16.5%, respectively). This ‘hexad’ cluster (with or without additional resistances) was found in 118 (19.0%) *E. coli* strains: 26.2% in chickens and 14.5% in humans (χ^2^ test, *P *< 0.001). The ‘pair’ cefpodoxime/ceftiofur was more common in humans (16.1%) than chickens (5.5%) isolates (*χ*^2^ test, *P *< 0.001). The cluster of resistance to azithromycin/cefpodoxime/ceftiofur was observed in 45 (10.2%) of all isolates; co-resistance to colistin and neomycin resistance was found in 6 isolates (0.9%) (Figure 2 and Table 2).

**Table 2.** Investigation of the level of phenotypic AMR matching between *E. coli* isolates from humans and chickens

Cluster	No. resistant isolates (%)			Matching between human and chicken *E. coli* (%)			Matching between human *E. coli* (%)			Matching between human *E. coli* (exposed to AMR *E. coli* in chicken)			Matching between human *E. coli* (non-exposed to AMR *E. coli* in chicken)		
	All (*n *= 622)	Chicken (*n *= 237)	Human (*n *= 385)	Same farms	Different farms	RR	Same farms	Different farms	RR	Same farms	Different farms	RR	Same farms	Different farms	RR
OTC	420 (67.5)	183 (77.2)	237 (61.6)	50.1	47.4	1.06*	40.4	38.6	1.05	41.7	40.9	1.02	34.4	29.3	1.17
DOX	389 (62.5)	180 (75.9)	209 (54.3)	42.6	41.1	1.04	32.4	30.0	1.08	31.6	30.5	1.04	36.1	28.0	1.29
AMX	378 (60.8)	163 (68.7)	215 (55.8)	41.3	38.4	1.07*	34.4	33.2	1.04	39.0	36.0	1.08	23.1	26.5	0.87
ENR	309 (49.7)	109 (51.1)	200 (51.9)	24.7	23.6	1.05	28.2	26.3	1.07	28.9	28.0	1.03	27.6	24.8	1.11
STR	282 (45.3)	121 (47.3)	161 (41.8)	20.0	21.2	0.94	18.1	17.8	1.02	18.1	14.6	1.24	18.1	21.1	0.86
FFN	215 (34.6)	112 (46.0)	103 (26.8)	15.3	12.8	1.19*	7.4	6.1	1.21	9.9	7.2	1.38	5.2	5.0	1.04
AZI	109 (17.5)	19 (8.0)	90 (23.4)	3.1	1.8	1.72*	7.4	6.4	1.16	15.0	11.8	1.27	6.5	5.8	1.12
POD	95 (15.3)	13 (5.5)	82 (21.3)	1.8	1.2	1.50	5.3	5.3	1.00	15.4	11.1	1.39	4.6	4.9	0.94
XNL	75 (12.1)	13 (5.5)	62 (16.1)	0.8	0.9	0.89	2.7	2.8	0.96	0	1.6	0	2.9	2.8	1.04
NEO	38 (6.1)	17 (7.2)	21 (5.5)	0.0	0.4	0.00	0.0	0.2	0.00	0	0	NC	0	0.3	0
COL	11 (1.8)	3 (1.3)	8 (2.1)	0.3	0.02	15.0	0.0	0.0	NC	0	0	NC	0	0	NC
NEO/COL	6 (0.9)	2 (0.8)	4 (1.0)	0.0	0.0	NC	0.0	0.0	NC	0	0	NC	0	0	NC
POD/XNL	75 (12.1)	13 (5.5)	62 (16.1)	0.8	0.9	0.89	2.7	2.8	0.96	0	1.6	0	2.9	2.8	1.04
DOX/OTC	386 (62.1)	179 (75.5)	207 (53.8)	42.3	40.5	1.04	31.4	29.3	1.07	30.9	30.0	1.03	33.3	26.3	1.27
POD/XNL/AZI	45 (7.2)	4 (1.7)	41 (10.6)	0.3	0.2	1.50	1.1	1.1	1.00	0	0	NC	1.1	1.1	1.0
DOX/OTC/AMX	313 (50.3)	153 (64.5)	160 (41.6)	30.4	26.7	1.14**	19.7	18.7	1.05	23.3	22.8	1.02	11.9	11.3	1.05
DOX/OTC/AMX/STR	225 (36.2)	107 (45.1)	118 (30.6)	14.8	13.6	1.09	9.6	9.8	0.98	13.0	10.4	1.25	8.2	9.3	0.88
DOX/OTC/AMX/STR/ENR	163 (26.2)	81 (34.2)	91 (23.6)	8.1	7.0	1.16	3.2	4.9	0.65	5.6	6.0	0.93	2.2	4.4	0.50
DOX/OTC/AMX/STR/ENR/FFN	118 (19.0)	62 (26.2)	56 (14.5)	4.7	3.8	1.24	1.6	1.7	0.94	4.0	2.3	1.74	0.7	1.5	0.47

OTC, oxytetracycline; DOX, doxycycline; AMX, amoxicillin; ENR, enrofloxacin; STR, streptomycin; FFN, florfenicol; AZI, azithromycin; POD, cefpodoxime; XNL, ceftiofur; NEO, neomycin; COL, colistin. NC, not calculated. RR, risk ratio. The significance of comparisons is indicated: **P < 0.01, *P < 0.05 [*P* values derived from randomization (20 000 iterations) of farm identity].

### Associations between AMU and AMR in human and chicken E. coli

**Figure 3.**
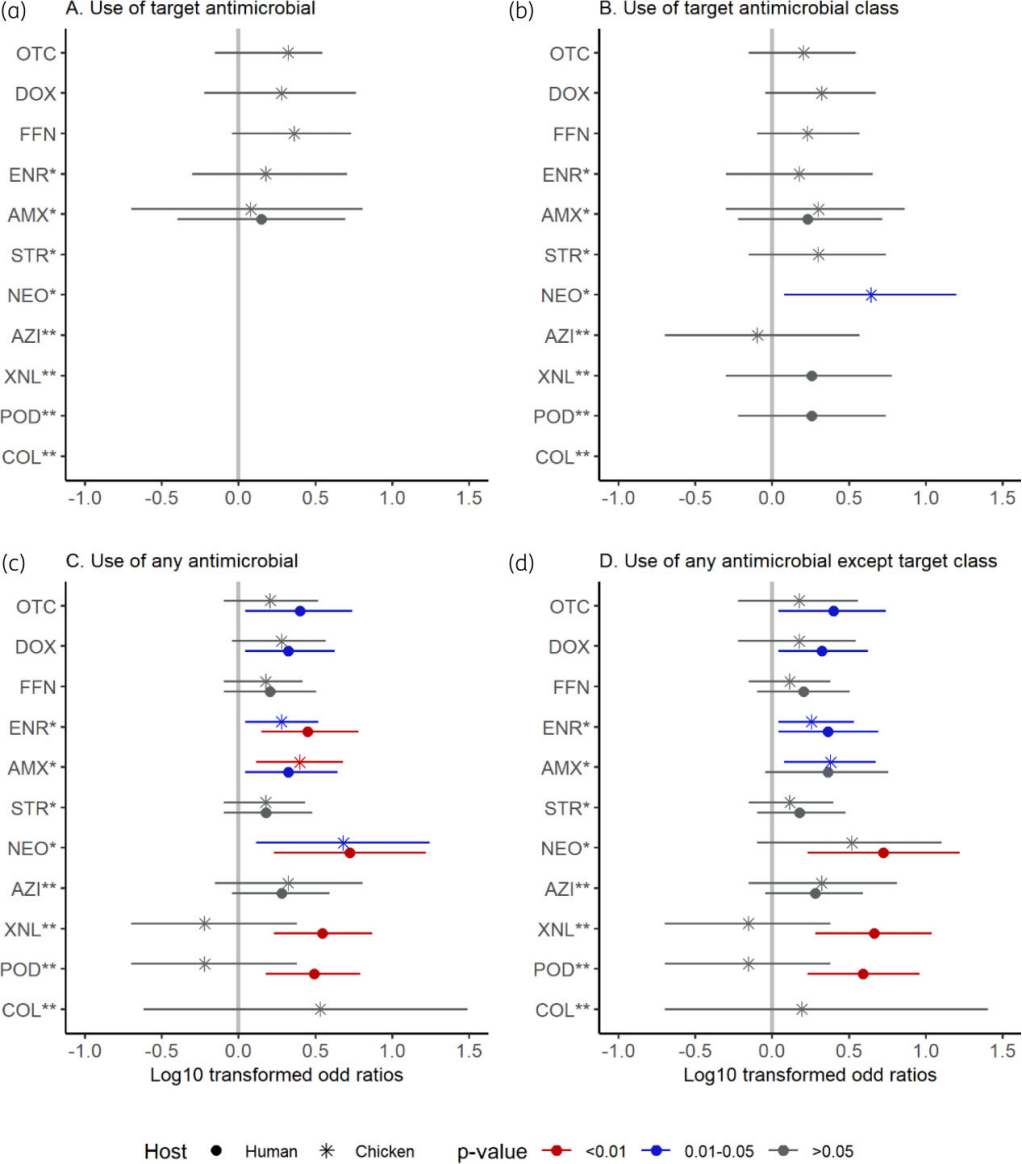
Log_10_-transformed odds ratios for AMR in human and chicken *E. coli* isolates. COL, colistin; POD, cefpodoxime; XNL, ceftiofur; AZI, azithromycin; NEO, neomycin; STR, streptomycin; AMX, amoxicillin; ENR, enrofloxacin; FFN, florfenicol; DOX, doxycycline; OTC, oxytetracycline. Critically important antimicrobials according to WHO (2018) are marked with ** (highest priority) and * (high priority).

Overall, the relationships between antimicrobial use and resistance were mostly positive for both human and chicken isolates (Figure 3). In humans, overall AMU increased the risk of resistance to a wide range of antimicrobials including neomycin [OR = 5.3, (95% CI, 1.7–16.6)], ceftiofur [OR = 3.5 (95% CI, 1.7–7.4)], cefpodoxime [OR = 3.1 (95% CI, 1.5–6.2)], enrofloxacin [OR = 2.8 (95% CI, 1.4–6.0)], oxytetracycline [OR = 2.5 (95% CI, 1.1–5.5)], amoxicillin [OR = 2.1 (95% CI, 1.1–4.4)] and doxycycline [OR = 2.1 (95% CI, 1.1–4.2)]. In chickens, there were strong associations between overall AMU and resistance to neomycin [OR = 4.8 (95% CI, 1.3–17.5)], amoxicillin [OR = 2.5, (95% CI, 1.3–4.8)] and enrofloxacin [OR = 1.9 (95% CI, 1.1–3.3)]. The use of aminoglycosides resulted in an increased risk of neomycin resistance [OR = 4.4 (95% CI, 1.2–15.8)]. When the target antimicrobial class was excluded, a contribution of AMU to resistance was still observed, except for amoxicillin and neomycin in human and chicken *E. coli* isolates, respectively.

The use of antimicrobials in animals (other than chickens) increased the risk of resistance to neomycin [OR = 3.7 (95%C I, 1.1–12.5)] and doxycycline [OR = 2.1 (95% CI, 1.1–4.4)] in humans (compared with those living in households without animals other than chickens present) (Table S10).

### Similarity of AMR between E. coli from chickens and humans in the same households

A total of 612 isolates from humans (*N *= 385) and chickens (*N *= 227) were investigated for similarity in their phenotypic AMR profile at the farm level.

The levels of similarity between human and chicken isolates with regards to oxytetracycline (RR = 1.06, *P *= 0.020), amoxicillin (RR = 1.07, *P *= 0.021), florfenicol (RR = 1.19, *P *= 0.022) and azithromycin (RR = 1.72, *P *= 0.035) resistance were higher for isolates coming from the same farms than those from different farms (Table 2). There was a suggestion of a higher probability of co-existence of colistin resistant *E. coli* in humans and chickens if they lived on the same farm (RR = 15.0, *P *= 0.102).

Among AMR clusters, *E. coli* from humans and chickens on the same farms showed a higher probability of carrying the doxycycline/oxytetracycline/amoxicillin resistance triad (30.4% versus 26.7%, *P *= 0.004) than those coming from different farms. Isolates from two hosts within-farm had a marginally higher degree of matching in their AMR patterns (of 11 tested antimicrobials) compared with a random sample of isolates from chickens and humans overall (*P *= 0.051, Figure S1).

The similarity of AMR clusters between human isolates were not affected by farm identity (Table 2). Furthermore, the level of matching AMR patterns (for 11 tested antimicrobials) of *E. coli* from humans living in the same households was not greater than the overall similarity between randomly selected human *E. coli* isolates (*P *= 0.469). Exposure to resistant *E. coli* from chickens may increase the probability of a matching cluster between in-farm human *E. coli* against streptomycin (RR = 1.24, *P *= 0.070), and florfenicol (RR = 1.38, *P *= 0.160).

## Discussion

Despite much higher AMU levels in chicken than in humans (291.1 per 1000 chicken-days versus 15.1 per 1000 person-days) and the set of tested antimicrobials based on the antimicrobials used in chicken production, we found surprisingly comparable levels of phenotypic AMR in *E. coli* from the two species. In accordance with a previous study in the area,^37^ we found a greater diversity of antimicrobials used in chickens (30 antimicrobials) than in humans (19 antimicrobials). Use of CIAs was observed in 50.9% of chicken flocks using antimicrobials. There were overlapping CIA classes (cephalosporins, macrolides, quinolones, and penicillins) used in humans and chickens, although the specific CIAs were largely different. The described levels of AMU in chickens are likely to be an underestimate since many of these flocks were raised on commercial feeds that contain antimicrobial growth promoters. However, we could not establish the composition of the feeds used by study flocks.

Our results demonstrated strong associations between AMU and AMR in both humans and chickens. Overuse and misuse of antimicrobials are important drivers of AMR, however, antimicrobial consumption alone does not explain the variations in AMR.^41^ Therefore, other factors in the environment need considering from One Health perspectives such as the presence and AMU of co-existing animals, occupation, diet, health, sanitation, and cultural traditions.^42^ A recent study showed that the association between AMU and AMR was more apparent for populations with higher hygiene levels.^43^

Our results suggest between-species transmission of phenotypic resistance to colistin, given the high probability of co-existence of colistin-resistant *E. coli* in humans and chickens on the same farms (RR = 15). It has been shown that exposure to *mcr*-1-positive chicken leads to increased risk of colonization by *mcr*-1-carrying bacteria in farmers.^25^ Vines *et al*.^44^ reported highly similar *mcr*-1-carrying plasmids (99% coverage, 99.97% identity) from *E. coli* of livestock and farmer origins although the isolates belonged to different lineages. Given the rare use of personal protective equipment when dealing with animals in rural farming systems,^25^ the overlapping of colistin resistance between human and animal bacteria could be attributed to exposure while farmers prepare the antimicrobials (powder) for their flocks.

We found greater matching of the doxycycline/oxytetracycline/amoxicillin resistance cluster between human and chicken samples from the same farms than between human and chicken samples from different farms, suggesting between-species transmission of this resistance phenotype. A potential explanation for this is the co-location of resistance genes on the same transferable genetic elements.^45^ Several studies have demonstrated the location of tetracycline and β-lactam resistance genes on the same plasmids (IncX1, IncI-1, IncFIB).^46,47^ Given the lack of use of protective equipment among farmers, as well as the great degree of overlap between the farm and living environments,^25^ the risks of transmission of resistant organisms and genetic determinants are likely to be high. However, overlapping patterns should be interpreted with caution since the direction of transmission is difficult to infer, and co-colonization from a shared source is also possible.^48^

This is one of the few studies describing the prevalence of AMR among healthy individuals in Vietnam. Compared with a study on healthy adults in Ho Chi Minh City, we observed a lower level of MDR among *E. coli* isolates (55.1% versus 71.8%) but higher resistance to third-generation cephalosporins (21.6% versus 15.5%).^22^ Interestingly, despite the high levels of AMR observed in that study, none of participants had a history of AMU in the previous 3 months. The observed prevalence of colistin resistance among chicken isolates (1.3%) was lower than previous reports (19%–22%) in this geographic area.^23,26^ This could be that chickens in this study were mostly sampled at a later stage of their cycle (median age of 27 weeks), while colistin is more commonly used during the brooding period.^19^ A recent study suggested that colistin resistance is short-lived in chicken flocks.^26^ Chickens are raised over litter (faecal matter) containing high bacterial loads and will be constantly exposed to new phenotypes; this may result in a greater challenge for the long-term persistence of resistance phenotypes (i.e. fitness costs) in that species.^49,50^ Colistin resistance among human isolates (2.1%) was lower than in a neighbouring province (Tien Giang) where 4.0% of *E. coli* from farmers carried the *mcr*-1 gene,^13^ but slightly higher than those in China, where 0.65% of healthy people carried *mcr*-1-harbouring *E. coli*.^51^

We acknowledge several limitations of our study. There could be the risks of false-positive findings due to multiple statistical testing. The cross-sectional study design limits the power to detect between-species transmission because resistant bacteria or genetic determinants may be absent at the time of sampling.^10^ The isolation of one *E. coli* isolate from each sample and the antimicrobial panel chosen (focused on antimicrobials used in chickens) may have reduced the chance of detecting relationships between AMU and resistance in the human species. Longitudinal studies based on a larger number of bacteria per subject and genetic traits would have been desirable. It is not possible to rule out that pooled chicken faecal samples may be contaminated by non-chicken *E. coli* present in the environment. Ideally, other environmental sources (i.e. wildlife, soil, and farm effluents) capable of infecting both host species should also have been investigated to fulfil the One Health study criteria,^52^ although this is extremely challenging.

In conclusion, using a One Health survey design that involved co-sampling of humans and chickens from same farms and integrated AMU and AMR data, we were able to demonstrate correlation between AMU and AMR, as well as potential interspecies transmission of certain resistance phenotypes.

## Supplementary Material

dlac054_Supplementary_Data

## Supplementary data

Tables S1 to S10 and Figure S1 are available as Supplementary data at *JAC* Online.
