# Supplementary material for: Antimicrobial resistance in commensal Escherichia coli from humans and chickens in the Mekong Delta of Vietnam is driven by antimicrobial usage and potential cross-species transmission
Source: JAC Antimicrob Resist. 2022 May 27;4(3):dlac054. doi: 10.1093/jacamr/dlac054 (PMC9154321; doi:10.1093/jacamr/dlac054)
Supplement: dlac054_Supplementary_Data [file dlac054_supplementary_data.zip › Suppl.Fig.docx]

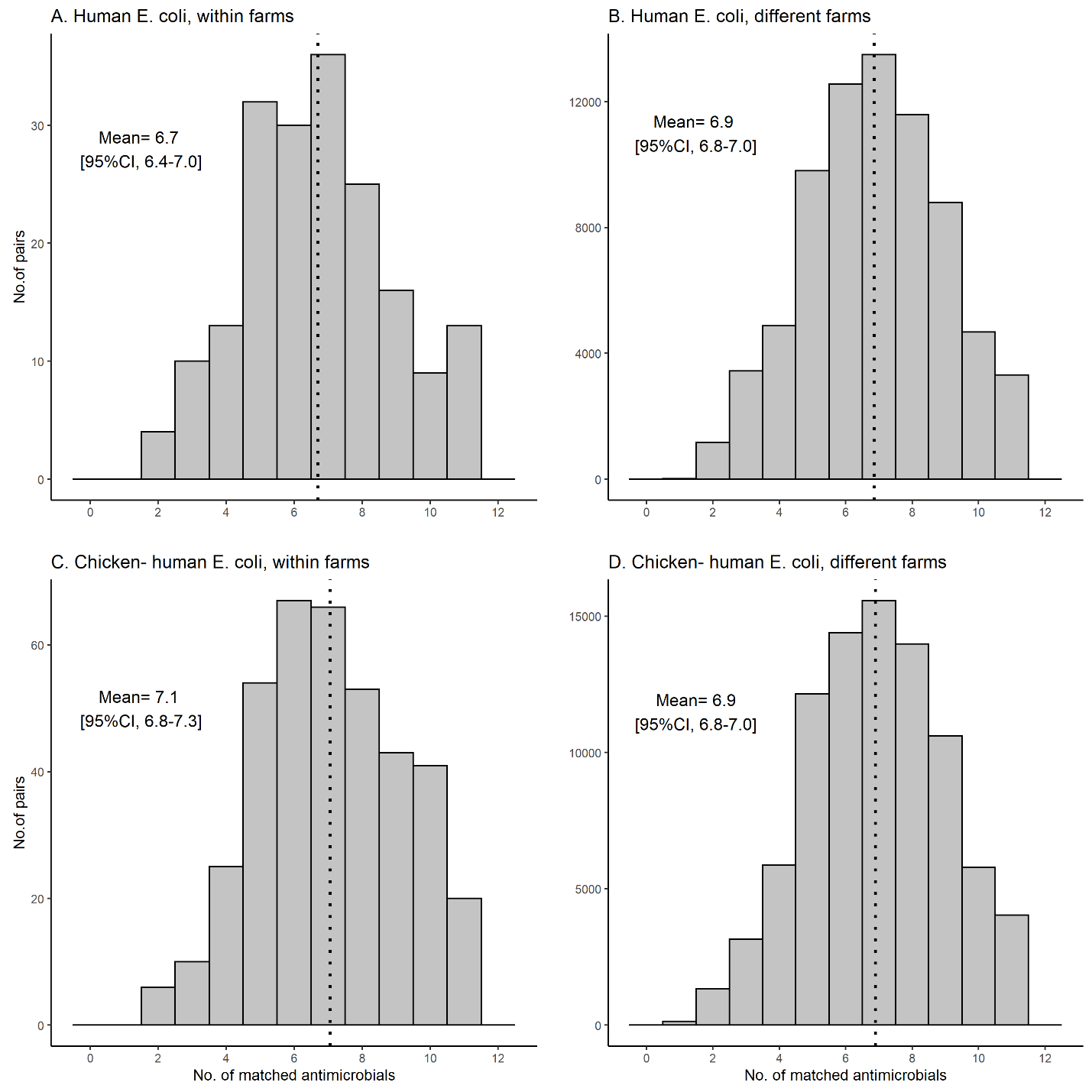


**Figure S1.** Number of matched AAIs among 11 test antimicrobials of *E. coli* isolates from (A) humans living in same farms, (B) humans living in different farms, (C) human and chicken living in same farms, (D) human and chicken living in different farms. Dotted lines indicate average number of matches.
