# Supplementary material for: Antimicrobial resistance in commensal Escherichia coli from humans and chickens in the Mekong Delta of Vietnam is driven by antimicrobial usage and potential cross-species transmission
Source: JAC Antimicrob Resist. 2022 May 27;4(3):dlac054. doi: 10.1093/jacamr/dlac054 (PMC9154321; doi:10.1093/jacamr/dlac054)
Supplement: dlac054_Supplementary_Data [file dlac054_supplementary_data.zip › Table S1.OxTREC503-20 Questionnaire for Farmer-animal.docx]

| **18ZNG – EXTENSION STUDY OF VIPARC PROJECT** |
| --- |
| **Questionnaire for farming households (Animal sample collection)** |

**Study: Engaging veterinary drug shops to reduce antimicrobial use and antimicrobial resistance
in the Mekong Delta of Vietnam**

*Oxford University Clinical Research Unit*

*Hospital for Tropical Diseases, Ho Chi Minh*

*Sub-Department of Animal Health and Production, Dong Thap*

*Centre of Disease Control Office, Dong Thap*

| **1** | Date of interview (dd/mm/yy) |  |
| --- | --- | --- |
| **2** | Initial name of interviewee |  |
| **3** | Farm ID |  |

| 1. **KNOWLEDGE** | | | |
| --- | --- | --- | --- |
| **1. Next, we will present you with the labels of 4 common veterinary products. We now would like you to guess which of the 5 is an antibiotic. Could you identify the product?** | | | |
| **2. Antibiotics are used to prevent bacterial diseases in farm animals?** | 🞎  True | 🞎  False | 🞎  Don’t know/Not sure |
| **3. Antibiotics are used to cure bacterial diseases in farm animals?** | 🞎  True | 🞎  False | 🞎  Don’t know/Not sure |
| **4. Antibiotics are used to promote growth in farm animals?** | 🞎  True | 🞎  False | 🞎  Don’t know/Not sure |
| **5. Antibiotics are used more often to treat humans rather than animals.** | 🞎  True | 🞎  False | 🞎  Don’t know/Not sure |
| **6. Antibiotic residues in meat and poultry can be transferred to humans.** | 🞎  True | 🞎  False | 🞎  Don’t know/Not sure |

| 1. **FARMING PRACTICE AND MEDICATION** | | | | | | | |
| --- | --- | --- | --- | --- | --- | --- | --- |
| **Livestock species present in household**  (select all that apply) | | | | **Medication use in the past 7 days** | | | |
| 🞎 Chicken | | | | 🞎 Yes | | 🞎 No/Don’t remember | |
| 🞎 Duck | | | | 🞎 Yes | | 🞎 No/Don’t remember | |
| 🞎 Muscovy duck | | | | 🞎 Yes | | 🞎 No/Don’t remember | |
| 🞎 Geese | | | | 🞎 Yes | | 🞎 No/Don’t remember | |
| 🞎 Quail | | | | 🞎 Yes | | 🞎 No/Don’t remember | |
| 🞎 Cattle | | | | 🞎 Yes | | 🞎 No/Don’t remember | |
| 🞎 Buffalo | | | | 🞎 Yes | | 🞎 No/Don’t remember | |
| 🞎 Goat/sheep | | | | 🞎 Yes | | 🞎 No/Don’t remember | |
| 🞎 Fish | | | | 🞎 Yes | | 🞎 No/Don’t remember | |
| 🞎 Shrimp | | | | 🞎 Yes | | 🞎 No/Don’t remember | |
| 🞎 Other (specify) | | | | 🞎 Yes | | 🞎 No/Don’t remember | |
| **Species** | |  | | | | | |
| **Age group**  (in months) | | **1**  [__ __] | **2**  [__ __] | | **3**  [__ __] | | **4**  [__ __] |
| **Number of animals** | |  |  | |  | |  |
| **Raising purpose** | |  |  | |  | |  |
| **Number of animals on medication**  (out of total) | | **__ __ /__ __** | **__ __ /__ __** | | **__ __ /__ __** | | **__ __ /__ __** |
| **Container/**  **record still available?** | Yes | 🞎 | 🞎 | | 🞎 | | 🞎 |
|  | No | 🞎 | 🞎 | | 🞎 | | 🞎 |
| **Antibiotics?** | Yes | 🞎 | 🞎 | | 🞎 | | 🞎 |
|  | No | 🞎 | 🞎 | | 🞎 | | 🞎 |
|  | Unknown | 🞎 | 🞎 | | 🞎 | | 🞎 |
| **If yes, duration?**  (out of 7 days) |  | **__ __ /**10 | **__ __ /**10 | | **__ __ /**10 | | **__ __ /**10 |
|  |  | **__ __ /**10 | **__ __ /**10 | | **__ __ /**10 | | **__ __ /**10 |
|  |  | **__ __ /**10 | **__ __ /**10 | | **__ __ /**10 | | **__ __ /**10 |
|  |  | **__ __ /**10 | **__ __ /**10 | | **__ __ /**10 | | **__ __ /**10 |
|  |  | **__ __ /**10 | **__ __ /**10 | | **__ __ /**10 | | **__ __ /**10 |
| **Reason**  (select all that apply) | If known, specify disease. Otherwise, select reason most closely applies | **___________________** | **___________________** | | **___________________** | | **___________________** |
|  | No disease/  prophylaxis | 🞎 | 🞎 | | 🞎 | | 🞎 |
|  | Noninfectious/ chronic diseases | 🞎 | 🞎 | | 🞎 | | 🞎 |
|  | Respiratory symptoms/  infections | 🞎 | 🞎 | | 🞎 | | 🞎 |
|  | Gastrointestinal symptoms/  Infections | 🞎 | 🞎 | | 🞎 | | 🞎 |
|  | Mouth and teeth symptoms/  infections | 🞎 | 🞎 | | 🞎 | | 🞎 |
|  | Wound/skin symptoms/  infections | 🞎 | 🞎 | | 🞎 | | 🞎 |
|  | Fever/general malaise symptoms/ infections | 🞎 | 🞎 | | 🞎 | | 🞎 |
|  | Other (specify) | 🞎 | 🞎 | | 🞎 | | 🞎 |
| **Whose advice?** | Drug seller | 🞎 | 🞎 | | 🞎 | | 🞎 |
|  | Vet/health professional | 🞎 | 🞎 | | 🞎 | | 🞎 |
|  | Family/friend | 🞎 | 🞎 | | 🞎 | | 🞎 |
|  | Personal experience | 🞎 | 🞎 | | 🞎 | | 🞎 |
|  | Other | 🞎 | 🞎 | | 🞎 | | 🞎 |

THANKS FOR YOUR PARTICIPATION
