# Supplementary material for: Antimicrobial resistance in commensal Escherichia coli from humans and chickens in the Mekong Delta of Vietnam is driven by antimicrobial usage and potential cross-species transmission
Source: JAC Antimicrob Resist. 2022 May 27;4(3):dlac054. doi: 10.1093/jacamr/dlac054 (PMC9154321; doi:10.1093/jacamr/dlac054)
Supplement: dlac054_Supplementary_Data [file dlac054_supplementary_data.zip › Table S2.OxTREC503-20 Questionnaire for Farmer-human.docx]

| **18ZNG – EXTENSION STUDY OF VIPARC PROJECT** |
| --- |
| **Questionnaire for farming households (human sample collection)** |

**Study: Engaging veterinary drug shops to reduce antimicrobial use and antimicrobial resistance
in the Mekong Delta of Vietnam**

*Oxford University Clinical Research Unit*

*Hospital for Tropical Diseases, Ho Chi Minh*

*Sub-Department of Animal Health and Production, Dong Thap*

*Centre of Disease Control Office, Dong Thap*

| **1** | Date of interview (dd/mm/yy) |  |
| --- | --- | --- |
| **2** | Initial name of interviewee |  |
| **3** | Farm ID |  |

| 1. **KNOWLEDGE (for household owner only)** | | | |
| --- | --- | --- | --- |
| **1. Have you ever heard of the term “antibiotics”?**  *If answer No or Don’t know/Not sure (code 99), proceed to part B.* | 🞎  Yes | 🞎  No | 🞎  Don’t know |
| **2. Next, we will present you with the labels of 4 common medicinal products. We now would like you to guess which of the 5 is an antibiotic. Could you identify the product?** | | | |
| **3. Antibiotics kill viruses?** | 🞎  True | 🞎  False | 🞎  Don’t know/Not sure |
| **4. Antibiotics are effective against colds and flu?** | 🞎  True | 🞎  False | 🞎  Don’t know/Not sure |
| **5. If you’re on a course of antibiotics, when can you stop?** | 🞎  When you feel better | 🞎  When you finished the course as directed | 🞎  Don’t know/Not sure |
| **6. It’s okay for your family members or friends/neighbors to use the same antibiotics you’ve used before, as long as they are for the same symptoms or illnesses.** | 🞎  True | 🞎  False | 🞎  Don’t know/Not sure |
| **7. It’s okay to keep or buy the same antibiotics from a doctor or pharmacist, if you’re sick and they helped you get better when you had the same symptoms before.** | 🞎  True | 🞎  False | 🞎  Don’t know/Not sure |

| **B. HOUSEHOLD AND ANTIBIOTIC USE INFORMATION** | | | | | | | | |
| --- | --- | --- | --- | --- | --- | --- | --- | --- |
| **Number of people in the household:** _____ | | | | | | | | |
| **Member** | | | **1** | **2** | **3** | **4** | **5** | **6** |
| **Age**  (in years, write 01 if < 1 year old) | | | [__ __] | [__ __] | [__ __] | [__ __] | [__ __] | [__ __] |
| **Gender** | Male | | 🞎 | 🞎 | 🞎 | 🞎 | 🞎 | 🞎 |
|  | Female | | 🞎 | 🞎 | 🞎 | 🞎 | 🞎 | 🞎 |
| **Education** | Did not attend | | 🞎 | 🞎 | 🞎 | 🞎 | 🞎 | 🞎 |
|  | Elementary | | 🞎 | 🞎 | 🞎 | 🞎 | 🞎 | 🞎 |
|  | Junior high school | | 🞎 | 🞎 | 🞎 | 🞎 | 🞎 | 🞎 |
|  | High school | | 🞎 | 🞎 | 🞎 | 🞎 | 🞎 | 🞎 |
|  | Some college | | 🞎 | 🞎 | 🞎 | 🞎 | 🞎 | 🞎 |
|  | University and above | | 🞎 | 🞎 | 🞎 | 🞎 | 🞎 | 🞎 |
| **Occupation**  **and yearly income** (select all that apply) | Farm-ing | Rice | 🞎  [_ _ _,_ _ _, _ _ _] | 🞎  [_ _ _,_ _ _, _ _ _] | 🞎  [_ _ _,_ _ _, _ _ _] | 🞎  [_ _ _,_ _ _, _ _ _] | 🞎  [_ _ _,_ _ _, _ _ _] | 🞎  [_ _ _,_ _ _, _ _ _] |
|  |  | Fruit | 🞎  [_ _ _,_ _ _, _ _ _] | 🞎  [_ _ _,_ _ _, _ _ _] | 🞎  [_ _ _,_ _ _, _ _ _] | 🞎  [_ _ _,_ _ _, _ _ _] | 🞎  [_ _ _,_ _ _, _ _ _] | 🞎  [_ _ _,_ _ _, _ _ _] |
|  |  | Live-stock | 🞎  [_ _ _,_ _ _, _ _ _] | 🞎  [_ _ _,_ _ _, _ _ _] | 🞎  [_ _ _,_ _ _, _ _ _] | 🞎  [_ _ _,_ _ _, _ _ _] | 🞎  [_ _ _,_ _ _, _ _ _] | 🞎  [_ _ _,_ _ _, _ _ _] |
|  | Small business | | 🞎  [_ _ _,_ _ _, _ _ _] | 🞎  [_ _ _,_ _ _, _ _ _] | 🞎  [_ _ _,_ _ _, _ _ _] | 🞎  [_ _ _,_ _ _, _ _ _] | 🞎  [_ _ _,_ _ _, _ _ _] | 🞎  [_ _ _,_ _ _, _ _ _] |
|  | General labor | | 🞎  [_ _ _,_ _ _, _ _ _] | 🞎  [_ _ _,_ _ _, _ _ _] | 🞎  [_ _ _,_ _ _, _ _ _] | 🞎  [_ _ _,_ _ _, _ _ _] | 🞎  [_ _ _,_ _ _, _ _ _] | 🞎  [_ _ _,_ _ _, _ _ _] |
|  | Public sector | | 🞎  [_ _ _,_ _ _, _ _ _] | 🞎  [_ _ _,_ _ _, _ _ _] | 🞎  [_ _ _,_ _ _, _ _ _] | 🞎  [_ _ _,_ _ _, _ _ _] | 🞎  [_ _ _,_ _ _, _ _ _] | 🞎  [_ _ _,_ _ _, _ _ _] |
|  | Private sector | | 🞎  [_ _ _,_ _ _, _ _ _] | 🞎  [_ _ _,_ _ _, _ _ _] | 🞎  [_ _ _,_ _ _, _ _ _] | 🞎  [_ _ _,_ _ _, _ _ _] | 🞎  [_ _ _,_ _ _, _ _ _] | 🞎  [_ _ _,_ _ _, _ _ _] |
|  | Other (specify) | | 🞎  [_ _ _,_ _ _, _ _ _] | 🞎  [_ _ _,_ _ _, _ _ _] | 🞎  [_ _ _,_ _ _, _ _ _] | 🞎  [_ _ _,_ _ _, _ _ _] | 🞎  [_ _ _,_ _ _, _ _ _] | 🞎  [_ _ _,_ _ _, _ _ _] |
| **Medication use in the past 3 months?** | Yes  No. of events | | 🞎  [__ __] | 🞎  [__ __] | 🞎  [__ __] | 🞎  [__ __] | 🞎  [__ __] | 🞎  [__ __] |
|  | No | | 🞎 | 🞎 | 🞎 | 🞎 | 🞎 | 🞎 |
|  | Don’t remember | | 🞎 | 🞎 | 🞎 | 🞎 | 🞎 | 🞎 |
| **Container/**  **record available?** | Yes | | 🞎 | 🞎 | 🞎 | 🞎 | 🞎 | 🞎 |
|  | No | | 🞎 | 🞎 | 🞎 | 🞎 | 🞎 | 🞎 |
| **Antibiotics?** (only complete following sections if answer is yes) | Yes  No. of use  (out of no. of events) | | 🞎  **__ __ /__ __** | 🞎  **__ __ /__ __** | 🞎  **__ __ /__ __** | 🞎  **__ __ /__ __** | 🞎  **__ __ /__ __** | 🞎  **__ __ /__ __** |
|  | No | | 🞎 | 🞎 | 🞎 | 🞎 | 🞎 | 🞎 |
|  | Unknown | | 🞎 | 🞎 | 🞎 | 🞎 | 🞎 | 🞎 |
| **If yes, duration?**  (product code in the blank, duration in days) |  | | [__ __] | [__ __] | [__ __] | [__ __] | [__ __] | [__ __] |
|  |  | | [__ __] | [__ __] | [__ __] | [__ __] | [__ __] | [__ __] |
|  |  | | [__ __] | [__ __] | [__ __] | [__ __] | [__ __] | [__ __] |
|  |  | | [__ __] | [__ __] | [__ __] | [__ __] | [__ __] | [__ __] |
|  |  | | [__ __] | [__ __] | [__ __] | [__ __] | [__ __] | [__ __] |
| **Reason** (select all that apply) | If known, specify disease. (Otherwise, select reason most closely applies) | | **_______________** | **_______________** | **_______________** | **_______________** | **_______________** | **_______________** |
|  | No disease/  Prophylaxis | | 🞎 | 🞎 | 🞎 | 🞎 | 🞎 | 🞎 |
|  | Non-  infectious/  chronic diseases | | 🞎 | 🞎 | 🞎 | 🞎 | 🞎 | 🞎 |
|  | Respiratory symptoms/  infections | | 🞎 | 🞎 | 🞎 | 🞎 | 🞎 | 🞎 |
|  | Gastro-  intestinal symptoms/  infections | | 🞎 | 🞎 | 🞎 | 🞎 | 🞎 | 🞎 |
|  | Mouth and teeth symptoms/  infections | | 🞎 | 🞎 | 🞎 | 🞎 | 🞎 | 🞎 |
|  | Wound/skin symptoms/  infections | | 🞎 | 🞎 | 🞎 | 🞎 | 🞎 | 🞎 |
|  | Fever/  general malaise symptoms/  infections | | 🞎 | 🞎 | 🞎 | 🞎 | 🞎 | 🞎 |
|  | Other (specify) | | 🞎 | 🞎 | 🞎 | 🞎 | 🞎 | 🞎 |
| **Whose advice?** | Drug seller | | 🞎 | 🞎 | 🞎 | 🞎 | 🞎 | 🞎 |
|  | Doctor/  health professional | | 🞎 | 🞎 | 🞎 | 🞎 | 🞎 | 🞎 |
|  | Family/  friend | | 🞎 | 🞎 | 🞎 | 🞎 | 🞎 | 🞎 |
|  | Personal experience | | 🞎 | 🞎 | 🞎 | 🞎 | 🞎 | 🞎 |
|  | Other | | 🞎 | 🞎 | 🞎 | 🞎 | 🞎 | 🞎 |

THANKS FOR YOUR PARTICIPATION
